# Direct Oral Anticoagulants vs. Vitamin K Antagonists in Atrial Fibrillation Patients at Risk of Falling: A Meta-Analysis

**DOI:** 10.3389/fcvm.2022.833329

**Published:** 2022-05-09

**Authors:** Xinxing Gao, Donghua Huang, Yuting Hu, Yuanyuan Chen, Haidong Zhang, Fuwei Liu, Jun Luo

**Affiliations:** ^1^Division of Cardiology, Department of Internal Medicine, People's Hospital of Zhuzhou, Changsha Medical University, Zhuzhou, China; ^2^Department of Cardiology, The Affiliated Ganzhou Hospital of Nanchang University, Ganzhou, China; ^3^Second Clinical Medical College, Nanchang University, Nanchang, China; ^4^First Clinical Medical College, Nanchang University, Nanchang, China; ^5^School of Medicine, Sun Yat-sen University, Shenzhen, China

**Keywords:** atrial fibrillation, fall, direct oral anticoagulants, warfarin, meta-analysis

## Abstract

**Background:**

Direct oral anticoagulants (DOACs) and warfarin are usually used for people with atrial fibrillation (AF). However, for the AF patients at risk of falling, the effectiveness and safety outcomes of DOACs compared with warfarin remain unclear. Therefore, we performed a meta-analysis regarding the effectiveness and safety of DOACs vs. warfarin in AF patients at risk of falling.

**Methods:**

A search of the PubMed and Embase databases until November 2021 was performed. We included studies if they satisfied the following criteria: (1) study type: randomized clinical trials or observational cohort studies. (2) Comparisons: effectiveness and/or safety of DOACs (dabigatran, rivaroxaban, apixaban, or edoxaban) compared with warfarin. (3) Study data: the sample size, the number of events in the VKAs or DOACs groups, adjusted risk ratios (RRs), and 95% confidence intervals (CIs). (4) Study outcomes: stroke or systemic embolism (SSE), ischemic stroke, myocardial infarction (MI), all-cause death, and cardiovascular death; major bleeding, major or clinically relevant non-major (CRNM) bleeding, intracranial bleeding, gastrointestinal bleeding, and any bleeding. (5) Study population: patients at risk of falling. According to the Morse Fall Scale, the risk of falling relates to the history of falling, secondary diagnosis, ambulatory aids, intravenous therapy, type of gait, and mental status. In this meta-analysis, if the patient's MFS score is ≥25 points, he will be thought of as having the risk of falling. The adjusted risk ratios (RRs) and 95% confidence intervals (CIs) were pooled by a random-effects model with an inverse variance method.

**Results:**

Three cohort studies were included in our study. For the effectiveness outcomes, the use of DOACs was only associated with a significantly reduced risk of hemorrhagic stroke (RR = 0.28, 95%CI:0.10–0.75) compared with warfarin, but there were no significant differences in stroke or systemic embolism (SSE) (RR = 0.87, 95%CI:0.70–1.08), cardiovascular death (RR = 0.97, 95%CI:0.73–1.29) and all-cause death (RR = 0.90, 95%CI:0.72–1.11). For the safety outcomes, the use of DOACs was significantly associated with reduced risks of major or clinically relevant non-major bleeding (RR = 0.77, 95%CI:0.61–0.98) and intracranial bleeding (RR = 0.26, 95%CI:0.11–0.66) but not major bleeding (RR = 0.78, 95%CI:0.58–1.06).

**Conclusions:**

Compared with warfarin, the use of DOACs in AF patients at risk of falling is significantly associated with reduced risks of hemorrhagic stroke, major or clinically relevant non-major bleeding, and intracranial bleeding.

## Introduction

As the most common arrhythmia, the incidence, and prevalence of atrial fibrillation have increased for the last 20 years and might keep this growing tendency in the next 30 years (1). And AF as an accompanying state is associated with a 1.5- to 1.9-fold mortality risk after adjustment for the former cardiovascular disease (2). Patients with AF have increased risks of death, stroke, heart failure (HF), and cognitive dysfunction (3), thus they have significantly poorer life quality compared with other patients with only coronary heart disease or healthy people (4). With the advancement of medical management and ablation procedures, AF hospitalization-related mortality has decreased from 7.5% in 2006 to 4.3% in 2015 (approximately by 42%), but hospital costs per year have increased exponentially by 468% during this 10 years period (5), which means AF has become one of the largest epidemic and public health problems in the world. Atrial fibrillation (AF) affects 60 million people worldwide (6), resulting in embolism events, deterioration of cardiac function, and a significant increase in overall mortality (7).

The incidence of AF rises as the age increases from 60-years-old, so does the risk of falling (8, 9). DOACs and warfarin are generally used in AF patients to prevent stroke. The advantage of DOACs over warfarin in reducing SSEs, hemorrhagic stroke, all-cause mortality, and intracranial hemorrhage has been studied by several previous studies (10). The risk of falling should not be a decisive factor for withholding anticoagulation as nowadays' anticoagulation guidelines seem to pay more attention to bleeding complications than the risk of stroke (11, 12). In addition, the evidence certifying the deleterious effects of warfarin and DOACs on bone health is insufficient (13). However, the effectiveness and safety outcomes comparing warfarin and DOACs use in AF patients at risk of falling are still unclear. Therefore, this meta-analysis aimed to compare the effectiveness and safety of DOACs with warfarin in AF patients at risk of falling.

## Methods

We performed this meta-analysis based on the protocol and reporting of the results from the PRISMA (Preferred Reporting Items for Systematic Reviews and Meta-Analyses) statement. We put the PRISMA 2020 Checklist in Supplementary Table 1.

### Literature Retrieval

PubMed and Embase were systematically searched until November 2021 for relevant studies through the following search terms: (atrial fibrillation) AND (non-vitamin K antagonist oral anticoagulants OR direct oral anticoagulants OR dabigatran OR rivaroxaban OR apixaban OR edoxaban) AND (vitamin K antagonists OR warfarin) AND (fall or falling). We applied no linguistic restrictions to the literature. The literature search strategy is shown in Supplementary Table 2.

### Inclusion and Exclusion Criteria

We included studies if they satisfied the following criteria: (1) study type: randomized clinical trials or observational cohort studies. (2) Comparisons: effectiveness and/or safety of DOACs (dabigatran, rivaroxaban, apixaban, or edoxaban) compared with warfarin. (3) Study data: the sample size, the number of events in the VKAs or DOACs groups, adjusted risk ratios (RRs), and 95% confidence intervals (CIs). (4) Study outcomes: stroke or systemic embolism (SSE), ischemic stroke, myocardial infarction (MI), all-cause death, and cardiovascular death; major bleeding, major or clinically relevant non-major (CRNM) bleeding, intracranial bleeding, gastrointestinal bleeding, and any bleeding. (5) Study population: patients at risk of falling. According to the Morse Fall Scale, the risk of falling relates to the history of falling, secondary diagnosis, ambulatory aids, intravenous therapy, type of gait, and mental status. In this meta-analysis, if the patient's MFS score is ≥25 points, he will be thought of as having the risk of falling (14). Studies were excluded if: (1) certain publication types such as reviews, case reports, case series, editorials, letters, and meeting abstract meta-analyses. (2) Studies with no sufficient data. (3) Studies with duplicate data.

### Study Selection and Data Extraction

Two authors (Hu Y.T. and Chen Y.Y.) screened all the retrieved studies by titles and abstracts firstly to find eligible studies. Then we read the full texts in more detail according to the inclusion and exclusion criteria. We solved the disagreements through discussion or consultation with another author. The data extraction is according to the standardized form. The following data of each study will be collected: the first author and publication year, study design, country, data source, follow-up time, patient age and sex, sample size, types of DOACs, effectiveness and safety outcomes used in the study, and adjusted risk ratios (RRs) and 95% confidence intervals (CIs).

### Study Quality Assessment

Because each included study belonged to a cohort study, we use the Newcastle–Ottawa Scale (NOS) tool to evaluate the quality. This scoring scale involved three domains: the selection of cohorts (0–4 points), the comparability of cohorts (0–2 points), and the assessment of the outcomes (0–3 points). A study with a NOS score of <6 was defined as low quality (15, 16).

### Statistical Analysis

We used the Manager Version 5.4 (The Nordic Cochrane Center, The Cochrane Collaboration, 2014, Copenhagen, Denmark; https://community.cochrane.org/) to conduct the statistical analysis. If a *P*-value is < 0.05, we admit it's statistically significant. The Cochrane Q-test and *I*^2^ statistic were chosen by us to evaluate consistency, in this way, a *P* < 0.1 for Q-test and *I*^2^ > 50% indicated a substantial heterogeneity. We calculated and pooled the natural logarithms of RRs and standard errors of the studies by a random-effects model using an inverse variance method. Firstly, the number of patients and events of two groups were collected to calculate corresponding crude effectiveness and safety outcomes rates. The results of DOACs or group warfarin groups were shown by odds ratios (ORs) and 95% CIs. Secondly, we used the adjusted RRs to further eliminate the influence of confounders and to evaluate the outcomes.

## Results

### Study Selection

The process of literature retrieval is shown in Supplementary Figure 2. A total of 111 studies were found through electronic searches. Removing the duplicate studies, 81 studies were used for the title/abstract screening. Then 13 remaining studies need to be assessed in more detail. Ten studies (11, 12, 17–24) were excluded because (1) mathematical model (*n* = 3); (2) case-control study (*n* = 1); (3) participants without AF (*n* = 1); (4) intervention is only warfarin (*n* =1); (5) studies without available data (*n* = 1); (6) studies' outcomes are the risk of falling (*n* = 2); (7) studies' outcomes are prescriptions (*n* = 1). Finally, a total of three cohort studies (25–27) were included in our meta-analysis.

### Baseline Characteristics of the Included Studies

The baseline characteristics of included studies are shown in Table 1. All of them are cohort studies and meet our inclusion criteria. For quality assessment, these three included studies had a moderate-to-high quality with a Newcastle-Ottawa Scale score of ≥6 points. The study by Rao et al. (25) included 753 patients with a mean age of 75 years old using apixaban in the DOACs therapy. The study by Steffel et al. (27) included 900 patients with the use of edoxaban at the mean age of 77. And the last study by Miao et al. (26) included a total of 25,144 patients receiving three kinds of DOACs (apixaban, edoxaban, and rivaroxaban) at the mean age of 83. More details of these included studies' characteristics are shown in Supplementary Table 4. Although the three cohort studies were performed in the US, it did not mean all the patients were Americans. In the study by Rao et al., the patients come from America, Europe and Asia. The study by Steffel et al. included multinational patients. Therefore, we didn't discuss the limitations or generalizability of the patient ethnicity. Therefore, we didn't discuss the limitations or generalizability of the patient ethnicity.

**Table 1:** Baseline characteristics of included studies.

**Included study**	**Study design**	**Country**	**Data source**	**Follow-up time (y)**	**Antiplatelet agents use rate**	**Sample size**	**Age, median (25th, 75th), years**	**Female sex, No. (%)**	**Definition of the risk of falling**	**DOACs**	**Safety outcomes**	**Effectiveness outcomes**	**Confounder**	**Warfarin-naïve or warfarin-users**
Rao et al. (25)	Cohort study	USA	Duke Clinical Research Institute	1.8	0%	753	75 (67, 79)	357 (47.4)	Patients with a history of falling	*Apixaban*	Major bleeding, major or CRNM bleeding and intracranial bleeding	SSE, cardiovascular death, all-cause death and hemorrhagic stroke	Comorbidities (e.g., cerebrovascular disease, peripheral vascular disease, congestive heart failure, prior MI), medication at randomization (ACE inhibitors/ARBs, Beta-blockers)	Unclear
Steffel et al. (27)	Cohort study	USA	ENGAGE AF–TIMI 48	2.8	0%	900	77 (72, 82)	445 (49.4)	Having any of the following eight criteria at randomization: 1) prior history of falls; 2) lower extremity weakness; 3) poor balance; 4) cognitive impairment; 5) orthostatic hypotension; 6) use of psychotropic drugs; 7) severe arthritis; or 8) dizziness.	*Edoxaban*	Major bleeding, major or CRNM bleeding and intracranial bleeding	SSE, cardiovascular death, all-cause death and hemorrhagic stroke	History of stroke or TIA, history of hypertension, history of coronary artery disease, history of coronary heart failure, aspirin use at randomization, dose reduced at randomization	Warfarin-naïve and warfarin-users
Miao et al. (26)	Cohort study	USA	United States (US) Truven MarketScan	1.4	18.0%	25,144	83(47.87)	10,297(41.0)	A predicted 2-year fall-risk ≥ 15% per the algorithm developed and validated by Homer et al.	*Apixaban, edoxabanandrivaroxaban*	Intracranial bleeding	SSE	Comorbidities (e.g., acute decompensated heart failure, genital urinary bleeding, ischemic stroke, cognitive artery bypass grafting, heart failure, coagulopathy) smoker, medication use like antiplatelet drugs	Warfarin-naïve

*DOACs, direct oral anticoagulants; CRNM, clinically relevant nonmajor bleeding; SSE, stroke or systemic embolism; NOS, Newcastle-Ottawa Scale*.

### Crude Event Rates Between DOACs and Warfarin

**Figure 1:**
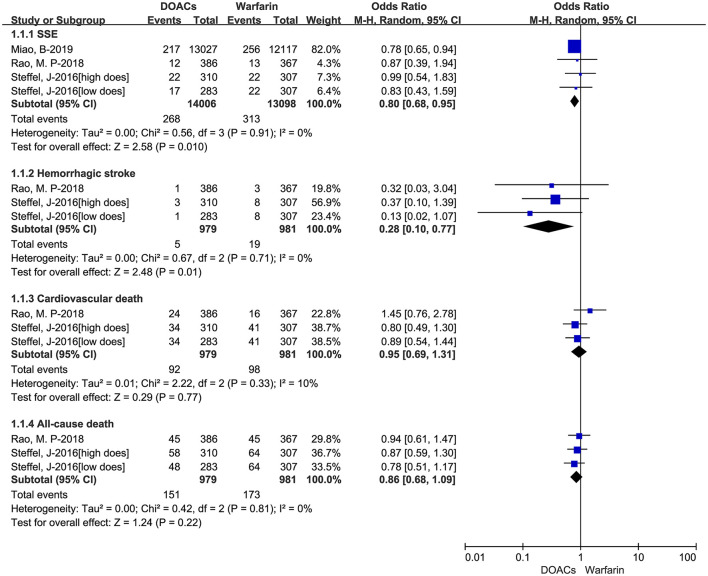
Crude effectiveness event rates of direct oral anticoagulants compared with warfarin among atrial fibrillation patients at risk of falling. DOACs, direct oral anticoagulants; SSE, stroke or systemic embolism.

**Figure 2:**
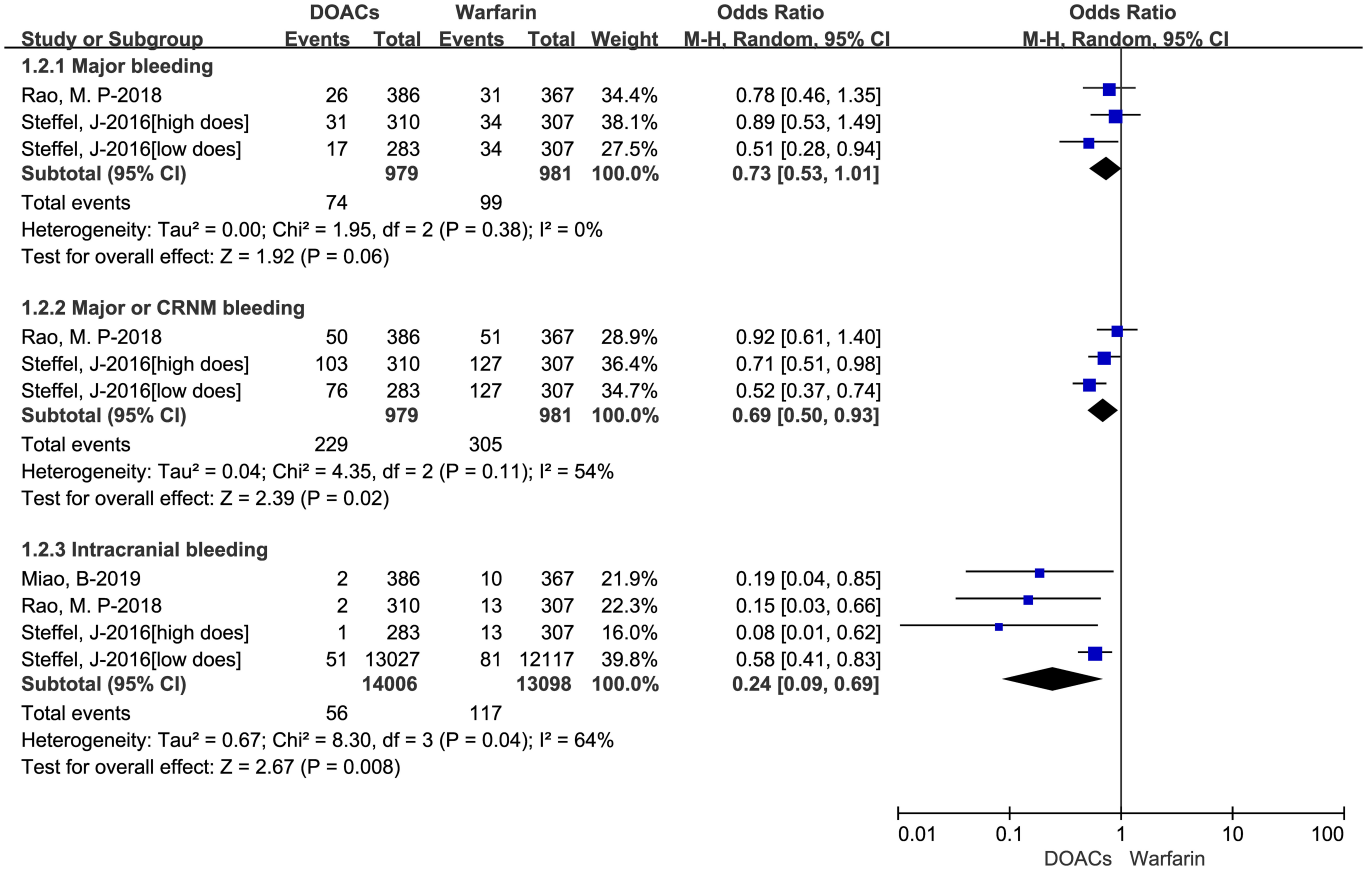
Crude safety event rates of direct oral anticoagulants compared with warfarin among atrial fibrillation patients at risk of falling. DOACs, direct oral anticoagulants; CRNM, clinically relevant nonmajor bleeding.

We put the effectiveness outcomes of three included studies in Figure 1. The use of DOACs is associated with lower event rates of SSE (1.91 vs. 2.39%, OR = 0.80, 95%CI:0.68–0.95), and hemorrhagic stroke (0.51 vs. 1.94%, OR = 0.28, 95%CI:0.10–0.77). But there were comparable rates of cardiovascular death (9.40 vs. 9.99%, OR = 0.95, 95%CI:0.69–1.31), and all-cause death (15.42 vs. 17.64%, OR = 0.86,95%CI:0.68–1.09). The safety outcomes of DOACs vs. warfarin are shown in Figure 2. Compared with warfarin-users, DOACs users had significantly lower event rates of major or CRNM bleeding (23.39 vs. 31.09%, OR = 0.69, 95%CI:0.50–0.93), and intracranial bleeding (0.40 vs. 0.89%, OR = 0.24, 95%CI:0.09–0.69). However, there were no significant differences in major bleeding (7.56 vs. 10.09%, OR = 0.73, 95%CI:0.53–1.01).

### Adjusted Data of Outcomes Between DOACs vs. Warfarin

**Figure 3:**
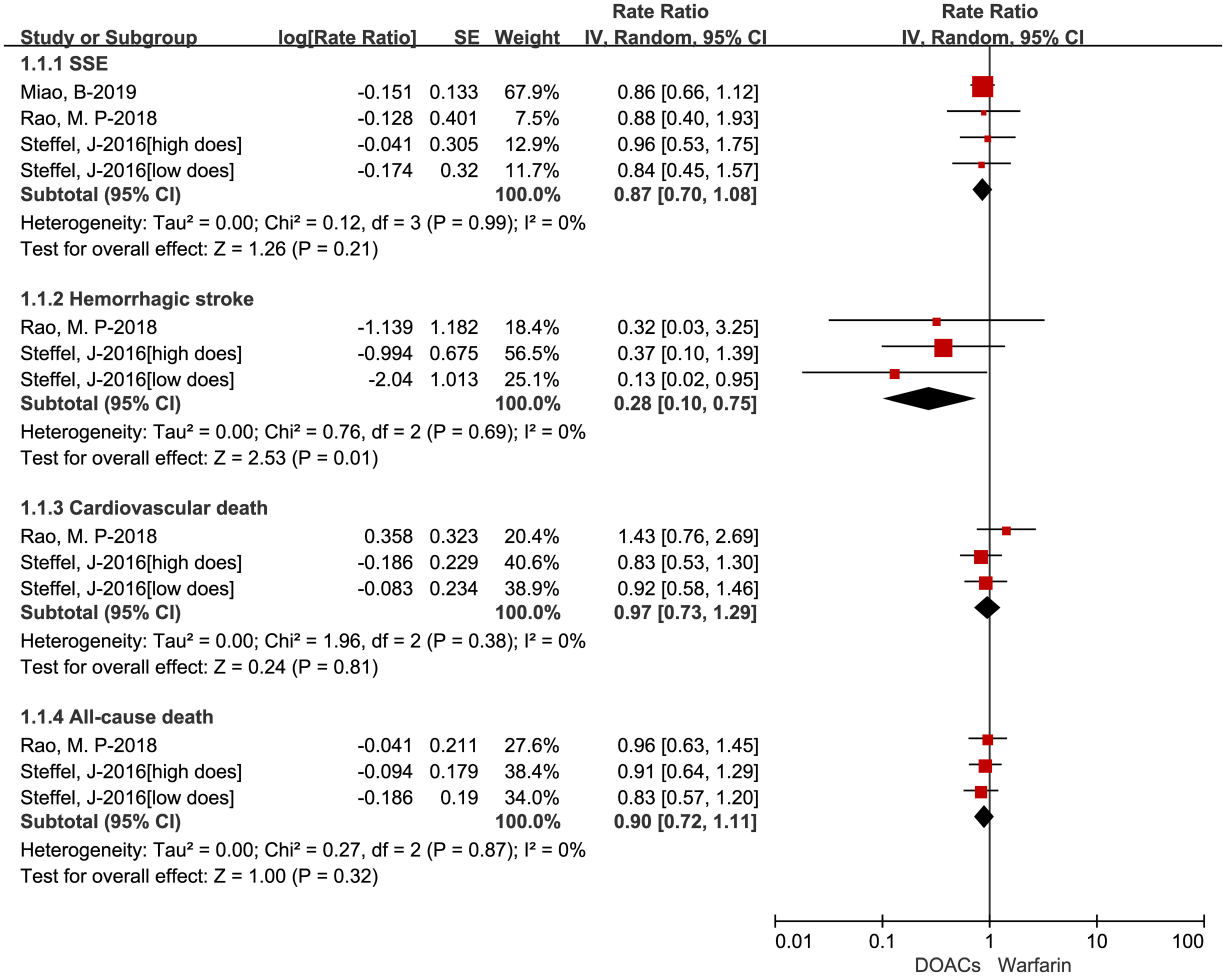
Adjusted effectiveness data of direct oral anticoagulants compared with warfarin among atrial fibrillation patients at risk of falling. DOACs, direct oral anticoagulants; SSE, stroke or systemic embolism.

**Figure 4:**
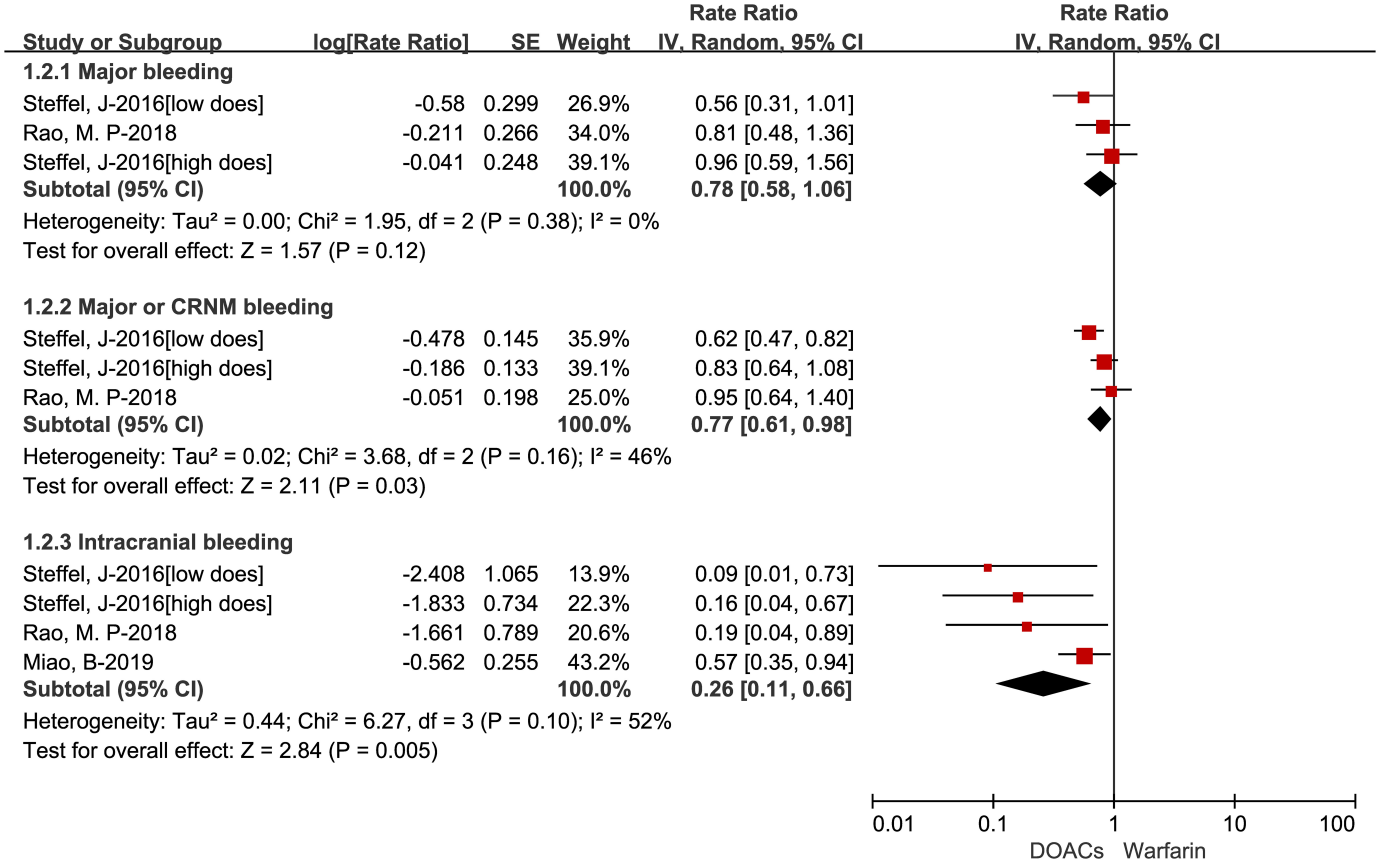
Adjusted safety data of direct oral anticoagulants compared with warfarin among atrial fibrillation patients at risk of falling. DOACs, direct oral anticoagulants; CRNM, clinically relevant nonmajor bleeding.

The adjusted data of effectiveness and safety outcomes among the three included studies are put in Figures 3, 4, respectively. For effectiveness outcomes, the use of DOACs was significantly associated with reduced risks of hemorrhagic stroke (RR = 0.28, 95%CI:0.10–0.75, *I* = 0%) compared with warfarin. But there were no significant differences in SSE (RR = 0.87, 95%CI:0.70–1.08, *I* = 0%), cardiovascular death (RR = 0.97, 95%CI:0.73–1.29, *I* = 0%), and all-cause death (RR = 0.90, 95%CI:0.72–1.11, *I* = 0%). For safety outcomes, users of DOACs had a significant association with decreased risks of major or CRNM bleeding (RR = 0.77, 95%CI:0.61–0.98, *I* = 46%) and intracranial bleeding (RR = 0.26, 95%CI:0.11–0.66, *I* = 52%) but not major bleeding (RR = 0.78, 95%CI:0.58–1.06, *I* = 0%) compared with warfarin-users.

Given the huge heterogeneity in sample size between the study by Miao et al. and the other two studies, we deleted the study and then assessed the effectiveness and safety of DOACs vs. VKAs in AF patients at risk of falling by using the adjusted RRs. The adjusted data of effectiveness and safety outcomes about the two included studies were presented in Supplementary Figures 3, 4. According to the Supplementary Figures 3, 4, if we deleted the Miao study, the outcomes turned to be stable.

### Publication Bias

Publication bias assessment should not be performed by the funnel plot approach when the number of studies is <10 because such meta-analyses are underpowered to detect such bias.

### Grade

The overall evidence for the observational studies was qualified using GRADE (grading of recommendations assessment, development, and evaluation) system categories. GRADE ratings of the quality of evidence in the three cohort studies are provided in Supplementary Table 5. According to GRADE system categories, the quality of evidence for outcomes we included was moderate.

## Discussion

The main findings of our meta-analysis are listed as follows: (1) The use of DOACs was significantly associated with reduced risk of hemorrhagic stroke compared with warfarin; (2) The use of DOACs resulted in significantly lower rates of major or CRNM bleeding and intracranial bleeding; (3) Compared with warfarin, DOACs showed comparable rates of SSE, cardiovascular death, all-cause death, intracranial bleeding, and major bleeding.

Due to the unique clinical features of AF patients at risk of falling, anticoagulation for these patients is contentious. For example, patients at high risk for falls with atrial fibrillation are at substantially increased risk of intracranial hemorrhage, especially traumatic intracranial hemorrhage (28). There was apparent underuse of anticoagulant therapy in AF patients at risk of falling, especially in the elderly, as there was inconsistency in opinion among clinicians on who should receive anticoagulation (29). Previous data suggested that physicians' decisions were guided more by their concerns over bleeding than an evaluation of the patient's risk of stroke (30). Global Anticoagulant Registry in the FIELD (GARFIELD) registry has demonstrated that falling risk and fear of bleeding are frequent reasons why clinicians chose to restrict anticoagulant therapy in AF patients despite guideline recommends anticoagulant therapy (31). In addition, some findings suggest that the risk of falling is not a valid reason to avoid oral anticoagulants in AF patients (32). Of note, European Society of Cardiology guidelines (33) did not suggest falling risk was an absolute contraindication to anticoagulation, but rather recommend withholding anticoagulation only in patients who experience “severe uncontrolled falls” such as those related to epilepsy and advanced multisystemic atrophic related-backward falls. Several studies have indicated an overall benefit from anticoagulation in AF patients at increased risk of falling, indicating that the risk of severe bleeding is counterbalanced by a similar reduction in the risk of stroke (27). Furthermore, the study by Acanfora et al. has been calculated that elderly patient should fall more than 300 times a year before overcoming the clinical benefit of oral anticoagulation (34).

Compared with warfarin, DOACs have more benefits. In terms of pharmacodynamics, DOACs might have natural advantages over warfarin since their mode of action does not affect factor VII and initiation of the coagulation cascade, with a potential reduction in the risk of bleeding in case of trauma, particularly intracranial bleeding. In addition, the shorter half-life time of DOACs might help to limit traumatic bleeding (35). Based on their ability to reduce the risk of intracranial hemorrhage compared with warfarin, oral factor Xa inhibitors like apixaban should be considered as strong alternatives to warfarin in AF patients deemed at higher risk of falling (26). Therefore, DOACs appear to have a better overall benefit-risk profile compared with warfarin (36). However, there are arguments regarding regular follow-up assessment in patients on DOACs, particularly the monitoring of relevant comorbidities such as renal failure, older age, or frailty (37) as DOACs exhibit predictable pharmacokinetic characteristics with fewer drug-drug interactions, which might reduce the need for routine coagulation monitoring and dose adjustment. Therefore, a part of physicians supported that extra attention and regular reviews are only required in elderly and frail patients to ensure safe and effective anticoagulation (38). Recent data displays that there has been an increase in the amount of newly diagnosed patients with AF at risk of stroke receiving guideline-recommended therapy since DOACs were introduced, predominantly driven by increased use of DOACs and reduced use of vitamin K antagonist (VKA) ± antiplatelet (AP) or AP alone (39). DOACs represented more than 60% of newly introduced anticoagulants in 2018. One study by Jurin et al. (40) anticipated that this trend of administrating DOACs would continue as the prices of these agents decline and as they become available for all patients' groups with indications for their use. Among the DOACs, apixaban is the preferred strategy from a public payer perspective for stroke prevention in older patients with atrial fibrillation and increased fall risk according to the health state transition model by Wong et al. (23).

The effectiveness and safety of DOACs compared with warfarin in AF patients at risk of falling have been explored in several recent studies. One systematic review by Grymonprez et al. (41) supported that the preserved efficacy and safety outcomes of apixaban and edoxaban in geriatric AF patients may warrant their use in this population prone to fall, especially because of the significantly lower intracranial bleeding risk. Unfortunately, it concluded only through two secondary analyses of phase III RCT studies. Besides this systematic review, no other systematic review or meta-analysis has been performed so far specifically comparing the effectiveness and safety of DOACs vs. warfarin in AF patients at risk of falling. Our meta-analysis was the largest and latest study comparing the effectiveness and safety outcomes of DOACs vs. warfarin in AF patients at risk of falling, potentially suggesting that DOACs might be considered more suitable for this special population.

## Limitations

First of all, only three cohort studies included in our meta-analysis, in the future, more studies will be added to confirm our findings. Secondly, the clinical characteristics of patients in different included studies were heterogeneous, because the probability of falling in each study was different and we didn't discuss the limitations or generalizability of the patient ethnicity. Thirdly, we ignored the different pharmacological properties and clinical effectiveness and safety of different DOACs, but regarding them together as one group, so we did not conduct a subgroup analysis of DOACs and warfarin in AF patients at risk of falling. Fourthly, the protocol of the systematic review and meta-analysis were not registered in PROSPERO. Fifthly, in the warfarin users, the time in the therapeutic range was not considered because the study Miao et al. didn't compare the NOACs vs. warfarin with a time in the therapeutic range ≥ 60%. Sixthly, one study included patients who were warfarin-naïve only, and one study included patients who were warfarin-naïve and warfarin-users, the last one was unclear. Finally, because we did not perform subgroup analysis based on whether or not antiplatelet drugs were used, in the future, we will study antiplatelet drugs for subgroup analysis.

## Conclusion

Based on our meta-analysis, AF patients at risk of falling using DOACs have a significant association with reduced risks of hemorrhagic stroke, major or CRNM bleeding, and intracranial bleeding.

## Data Availability Statement

The original contributions presented in the study are included in the article/Supplementary Material, further inquiries can be directed to the corresponding authors.

## Author Contributions

All authors listed have made a substantial, direct, and intellectual contribution to the work and approved it for publication.

## Conflict of Interest

The authors declare that the research was conducted in the absence of any commercial or financial relationships that could be construed as a potential conflict of interest.

## Publisher's Note

All claims expressed in this article are solely those of the authors and do not necessarily represent those of their affiliated organizations, or those of the publisher, the editors and the reviewers. Any product that may be evaluated in this article, or claim that may be made by its manufacturer, is not guaranteed or endorsed by the publisher.

## References

[1] Lippi G Sanchis-Gomar F Cervellin G. Global epidemiology of atrial fibrillation: an increasing epidemic and public health challenge. Int J Stroke. (2021) 16:217–21. 10.1177/1747493019897870 31955707

[2] Benjamin EJ Wolf PA D'Agostino RB Silbershatz H Kannel WB Levy D. Impact of atrial fibrillation on the risk of death: the Framingham Heart Study. Circulation. (1998) 98:946–52. 10.1161/01.CIR.98.10.946 9737513

[3] Blum S Meyre P Aeschbacher S Berger S Auberson C Briel M . Incidence and predictors of atrial fibrillation progression: a systematic review and meta-analysis. Heart Rhythm. (2019) 16:502–10. 10.1016/j.hrthm.2018.10.022 30366160

[4] Thrall G Lane D Carroll D Lip GY. Quality of life in patients with atrial fibrillation: a systematic review. Am J Med. (2006) 119:448.e1–19. 10.1016/j.amjmed.2005.10.057 16651058

[5] Ribeiro AL Otto CM. Heartbeat: the worldwide burden of atrial fibrillation. Heart. (2018) 104:1987–8. 10.1136/heartjnl-2018-314443 30482808

[6] Global Global burden of 369 diseases and injuries in 204 countries and territories 1990–2019: 1990–2019: a systematic analysis for the Global Burden of Disease Study 2019. Lancet. (2020) 396:1204–22. 10.1016/S0140-6736(20)30925-9 33069326 PMC7567026

[7] Bassand JP Virdone S Goldhaber SZ Camm AJ Fitzmaurice DA Fox K . Early risks of death, stroke/systemic embolism, and major bleeding in patients with newly diagnosed atrial fibrillation. Circulation. (2019) 139:787–98. 10.1161/CIRCULATIONAHA.118.035012 30586740

[8] Singer DE Albers GW Dalen JE Go AS Halperin JL Manning WJ. Antithrombotic therapy in atrial fibrillation: the Seventh ACCP Conference on Antithrombotic and Thrombolytic Therapy. Chest. (2004) 126(3 Suppl):429S−56S. 10.1378/chest.126.3_suppl.429S 15383480

[9] Summary of the updated American Geriatrics Society/British Geriatrics Society Clinical Practice guideline for prevention of falls in older persons. J Am Geriatr Soc. (2011) 59:148–57. 10.1111/j.1532-5415.2010.03234.x 21226685

[10] Ruff CT Giugliano RP Braunwald E Hoffman EB Deenadayalu N Ezekowitz MD . Comparison of the efficacy and safety of new oral anticoagulants with warfarin in patients with atrial fibrillation: a meta-analysis of randomised trials. Lancet. (2014) 383:955–62. 10.1016/S0140-6736(13)62343-0 24315724

[11] Obi CA Bulsara K Izard S Delicce A Smith A Kim EJ. Examination of anticoagulation prescription among elderly patients with atrial fibrillation after in-hospital fall. J Thromb Thrombolysis. (2021) 9:10. 10.1007/s11239-021-02555-8 34480676

[12] Wei W Rasu RS Hernández-Muñoz JJ Flores RJ Rianon NJ Hernández-Vizcarrondo GA . Impact of fall risk and direct oral anticoagulant treatment on quality-adjusted life-years in older adults with atrial fibrillation: a Markov decision analysis. Drugs Aging. (2021) 38:713–23. 10.1007/s40266-021-00870-6 34235644

[13] Sugiyama T Oda H. Edoxaban versus Warfarin: bone fractures due to falling. J Am Coll Cardiol. (2017) 69:466–7. 10.1016/j.jacc.2016.10.071 28126164

[14] Schwendimann R De Geest S Milisen K. Evaluation of the Morse Fall Scale in hospitalised patients. Age Ageing. (2006) 35:311–3. 10.1093/ageing/afj066 16527829

[15] Zhu W Ye Z Chen S Wu D He J Dong Y . Comparative effectiveness and safety of non-vitamin K antagonist oral anticoagulants in atrial fibrillation patients. Stroke. (2021) 52:1225–33. 10.1161/STROKEAHA.120.031007 33596677

[16] Zhou Y Ma J Zhu W. Efficacy and safety of direct oral anticoagulants versus Warfarin in patients with atrial fibrillation across BMI categories: a systematic review and meta-analysis. Am J Cardiovasc Drugs. (2020) 20:51–60. 10.1007/s40256-019-00362-4 31342343

[17] Dodson JA Petrone A Gagnon D Tinetti M Krumholz H Gaziano J. Incidence and determinants of fall-related major bleeding among older adults with atrial fibrillation. (2015) 63:S6. 10.1001/jamacardio.2015.0345 27437657 PMC5600874

[18] Faramarzi N Sohal S Tsourkas P Al-Azzam B Cattoni HJM Rajabirostami E . In hospital outcome of elderly patients admitted with fall who have been anticoagulated, warfarin versus noacs? (2018) 13:10. Available online at: https://shmabstracts.org/abstract/in-hospital-outcome-of-elderly-patients-admitted-with-fall-who-have-been-anticoagulated-warfarin-versus-noacs/

[19] Fermann GJ James R Moore K Shah B Hylek E Granger C . Readmissions for major bleeding or falls are uncommon in an unselected population of patients hospitalized with atrial fibrillation. (2018) 71. 10.1016/S0735-1097(18)31034-9

[20] Jurin I Lucijanić M Radonić V Letilović T Lucijanić J Mesarov S . The risk of falling and consequences of falling in patients with atrial fibrillation receiving different types of anticoagulant. Drugs Aging. (2021) 38:417–25. 10.1007/s40266-021-00843-9 33650035

[21] Lin KJ Pawar A Gautam N Kim DH. Predicting fall risks in older adults with atrial fibrillation. (2020) 29(SUPPL 3):297–8. 10.1002/pds.5114 33058357

[22] Sorah AB Cunningham K Morgan JT Rinaldi M Christmas AB Sing R. Stroke risk versus fall risk: a growing conundrum in the anticoagulation of geriatric patients with atrial fibrillation. (2019) 73:1868. 10.1016/S0735-1097(19)32474-X

[23] Wong EKC Belza C Naimark DMJ Straus SE Wijeysundera HC. Cost-effectiveness of antithrombotic agents for atrial fibrillation in older adults at risk for falls: a mathematical modelling study. CMAJ Open. (2020) 8:E706−14. 10.9778/cmajo.20200107 33158928 PMC7661050

[24] Wong EK Kosar C Wijeysundera H. Markov decision analysis of the cost-utility for anticoagulant therapy in patients with atrial fibrillation at risk for falls. (2019) 34:S279.

[25] Rao MP Vinereanu D Wojdyla DM Alexander JH Atar D Hylek EM . Clinical outcomes and history of fall in patients with atrial fibrillation treated with oral anticoagulation: insights from the Aristotle trial. Am J Med. (2018) 131:269–75.e2. 10.1016/j.amjmed.2017.10.036 29122636

[26] Miao B Alberts MJ Bunz TJ Coleman CI. Safety and effectiveness of oral factor Xa inhibitors versus warfarin in nonvalvular atrial fibrillation patients at high-risk for falls. J Thromb Thrombolysis. (2019) 48:366–72. 10.1007/s11239-019-01898-7 31228038

[27] Steffel J Giugliano RP Braunwald E Murphy SA Mercuri M Choi Y . Edoxaban versus warfarin in atrial fibrillation patients at risk of falling: ENGAGE AF-TIMI 48 analysis. J Am Coll Cardiol. (2016) 68:1169–78. 10.1016/j.jacc.2016.06.034 27609678

[28] Gage BF Birman-Deych E Kerzner R Radford MJ Nilasena DS Rich MW. Incidence of intracranial hemorrhage in patients with atrial fibrillation who are prone to fall. Am J Med. (2005) 118:612–7. 10.1016/j.amjmed.2005.02.022 15922692

[29] Garwood CL Corbett TL. Use of anticoagulation in elderly patients with atrial fibrillation who are at risk for falls. Ann Pharmacother. (2008) 42:523–32. 10.1345/aph.1K498 18334606

[30] Sellers MB Newby LK. Atrial fibrillation, anticoagulation, fall risk, and outcomes in elderly patients. Am Heart J. (2011) 161:241–6. 10.1016/j.ahj.2010.11.002 21315204

[31] Kakkar AK Mueller I Bassand JP Fitzmaurice DA Goldhaber SZ Goto S . Risk profiles and antithrombotic treatment of patients newly diagnosed with atrial fibrillation at risk of stroke: perspectives from the international, observational, prospective GARFIELD registry. PLoS ONE. (2013) 8:e63479. 10.1371/journal.pone.0063479 23704912 PMC3660389

[32] Donzé J Clair C Hug B Rodondi N Waeber G Cornuz J . Risk of falls and major bleeds in patients on oral anticoagulation therapy. Am J Med. (2012) 125:773–8. 10.1016/j.amjmed.2012.01.033 22840664

[33] Kirchhof P Benussi S Kotecha D Ahlsson A Atar D Casadei B . 2016 ESC guidelines for the management of atrial fibrillation developed in collaboration with EACTS. Eur Heart J. (2016) 37:2893–962. 10.1093/eurheartj/ehw210 27567408

[34] Acanfora D Ciccone MM Scicchitano P Ricci G Acanfora C Uguccioni M . Efficacy and safety of direct oral anticoagulants in patients with atrial fibrillation and high thromboembolic risk. A systematic review. Front Pharmacol. (2019) 10:1048. 10.3389/fphar.2019.01048 31607911 PMC6761253

[35] Hylek EM Ko D. Atrial fibrillation and fall risk: what are the treatment implications? J Am Coll Cardiol. (2016) 68:1179–80. 10.1016/j.jacc.2016.07.714 27609679

[36] Hindricks G Potpara T Dagres N Arbelo E Bax JJ Blomström-Lundqvist C . 2020 ESC Guidelines for the diagnosis and management of atrial fibrillation developed in collaboration with the European Association for Cardio-Thoracic Surgery (EACTS): the Task Force for the diagnosis and management of atrial fibrillation of the European Society of Cardiology (ESC) Developed with the special contribution of the European Heart Rhythm Association (EHRA) of the ESC. Eur Heart J. (2021) 42:373–498. 10.1093/eurheartj/ehaa612 32860505

[37] Steffel J Verhamme P Potpara TS Albaladejo P Antz M Desteghe L . The 2018 European Heart Rhythm Association Practical Guide on the use of non-vitamin K antagonist oral anticoagulants in patients with atrial fibrillation. Eur Heart J. (2018) 39:1330–93. 10.1093/eurheartj/ehy136 29562325

[38] Bauersachs RM Herold J. Oral anticoagulation in the elderly and frail. Hamostaseologie. (2020) 40:74–83. 10.1055/s-0040-1701476 32000266

[39] Camm AJ Accetta G Ambrosio G Atar D Bassand JP Berge E . Evolving antithrombotic treatment patterns for patients with newly diagnosed atrial fibrillation. Heart. (2017) 103:307–14. 10.1136/heartjnl-2016-309832 27647168 PMC5293840

[40] Jurin I Lucijanić M Šakić Z Hulak KV Atić A Magličić A . Patterns of anticoagulation therapy in atrial fibrillation: results from a large real-life single-center registry. Croat Med J. (2020) 61:440–9. 10.3325/cmj.2020.61.440 33150762 PMC7684544

[41] Grymonprez M Steurbaut S De Backer TL Petrovic M Lahousse L. Effectiveness and safety of oral anticoagulants in older patients with atrial fibrillation: a systematic review and meta-analysis. Front Pharmacol. (2020) 11:583311. 10.3389/fphar.2020.583311 33013422 PMC7509201

